# Serum Adipocyte Fatty Acid-Binding Protein Level is Negatively Associated with Vascular Reactivity Index Measured by Digital Thermal Monitoring in Kidney Transplant Patients

**DOI:** 10.3390/metabo9080159

**Published:** 2019-07-31

**Authors:** Tai-Li Chen, Ming-Che Lee, Ching-Chung Ho, Bang-Gee Hsu, Jen-Pi Tsai

**Affiliations:** 1 School of Medicine, Tzu Chi University, Hualien 97004, Taiwan; 2 Department of Surgery, Hualien Tzu Chi Hospital, Buddhist Tzu Chi Medical Foundation, Hualien 97004, Taiwan; 3 Division of Nephrology, Hualien Tzu Chi Hospital, Buddhist Tzu Chi Medical Foundation, Hualien 97004, Taiwan; 4 Division of Nephrology, Department of Internal Medicine, Dalin Tzu Chi Hospital, Buddhist Tzu Chi Medical Foundation, Chiayi 62247, Taiwan

**Keywords:** adipocyte fatty acid-binding protein, digital thermal monitoring test, endothelial function, vascular reactivity index, kidney transplantation

## Abstract

Adipocyte fatty acid-binding protein (A-FABP) is abundantly found in mature adipocytes and is involved in cardiovascular disease. Our aim is to investigate the association between serum A-FABP levels and endothelial function among kidney transplant (KT) patients. Fasting blood samples were obtained from 80 KT patients. Serum A-FABP levels were measured using a commercially available enzyme immunoassay kit. Endothelial function and vascular reactivity index (VRI) were measured using digital thermal monitoring test. In this study, VRI < 1.0, VRI 1.0–1.9, and VRI ≥ 2.0 were defined as poor, intermediate, and good vascular reactivity, respectively. There were 12 (15.0%), 30 (37.5%), and 38 (47.5%) KT patients categorized as having poor, intermediate, and good vascular reactivity, respectively. Increased serum levels of alkaline phosphatase (*p* = 0.012), γ-glutamyltranspeptidase (GGT; *p* = 0.032), and A-FABP (*p* < 0.001) were associated with decreased vascular reactivity. Multivariable forward stepwise linear regression analysis revealed that age (β = −0.283, adjusted R^2^ change = 0.072; *p* = 0.003) and serum log-A-FABP level (β = −0.514, adjusted R^2^ change = 0.268; *p* < 0.001) were significantly associated with VRI values in KT patients. We concluded that serum fasting A-FABP level is negatively associated with VRI values and plays a role in endothelial dysfunction of KT patients.

## 1. Introduction

The risk of cardiovascular diseases (CVDs) in patients with a functioning graft remains higher than that in the general population. Therefore, determining CV risk factors among patients after kidney transplant (KT) may lead to risk modification to improve survival [1]. Despite recent improvements in techniques and medications for KT, endothelial dysfunction inevitably occurs in KT patients, with the endothelium being injured by ischemia–reperfusion, pharmacological agents, circulating inflammatory cells, cytokines, and antibodies [2]. Therefore, assessing endothelium function using biomarkers or non-invasive methods has been a hot topic of study in monitoring CV health of and predicting mortality in KT patients [3,4,5,6].

Fatty acid-binding proteins (FABPs), a family of 14–15 kDa intracellular chaperones that bind to long-chain fatty acids and other lipids, were first identified in 1972 [7]. Adipocyte fatty acid-binding protein (A-FABP) is a 14.6-kDa polypeptide with 132 amino acids, known as FABP4 or adipocyte P2; it is a member of the FABP family and is expressed in adipocytes, dendritic cells, and macrophages [8]. Additionally, it was thought to be involved in insulin resistance, chronic inflammation, and atherosclerotic processes [8]. The expression of A-FABP is regulated according to adipocyte differentiation and induced by peroxisome proliferator-activated receptor γ agonists, oxidized low-density lipoprotein, and advanced glycation end products [9]. Previous reports demonstrated that an increase in circulating A-FABP level was associated with several metabolic disorders and CVDs, such as obesity, diabetes mellitus (DM), hypertension (HTN), and cardiac dysfunction [10,11,12]. Serum A-FABP concentration is higher in critically ill patients and could thus be a prognostic factor for complications and mortality [13]. Several recent studies reported that circulating A-FABP served not only as a biomarker but also as an adipokine in the processing of metabolic syndrome and CV events [14]. In addition, we recently reported a positive correlation between serum A-FABP levels and metabolic syndrome in KT patients [15]. Given that CVDs are a major cause of mortality in the KT population, and adipokines could play an important role, we aimed to examine risk factors for endothelial function measured by digital thermal monitoring test and the association between serum A-FABP levels and endothelial function among KT patients.

## 2. Results

Clinical characteristics and immunosuppressive drugs used in KT patients are presented in Table 1. Of the 80 KT patients, 12 (15.0%), 30 (37.5%), and 38 (47.5%) were individually diagnosed as having poor, intermediate, and good vascular reactivity index (VRI), respectively. Serum alkaline phosphatase (ALP; *p* = 0.012), γ-glutamyltranspeptidase (GGT; *p* = 0.032), and serum A-FABP levels (*p* < 0.001) were significantly increased as VRI decreased among KT patients. Of these KT patients, 39 (48.8%) had DM and 31 (38.8%) had HTN. The most commonly prescribed immunosuppressive agents were tacrolimus (*n* = 53; 60.3%), cyclosporine (*n* = 14; 17.5%), mycophenolate mofetil (*n* = 48; 60.0%), steroids (*n* = 68; 85.0%), and rapamycin (*n* = 8; 10.0%). There were no significant differences in sex, modes of transplantation, presence of DM or HTN, or the use of immunosuppression medications among the three VRI groups.

**Figure 1:**
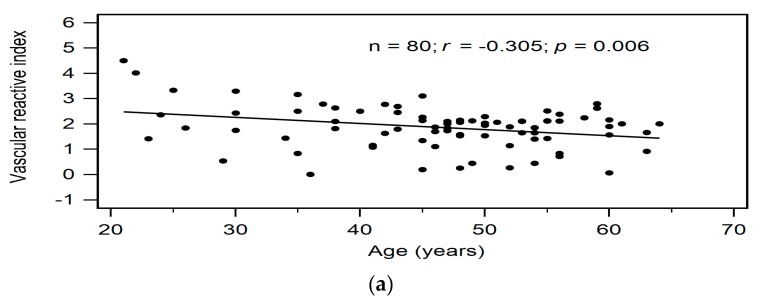

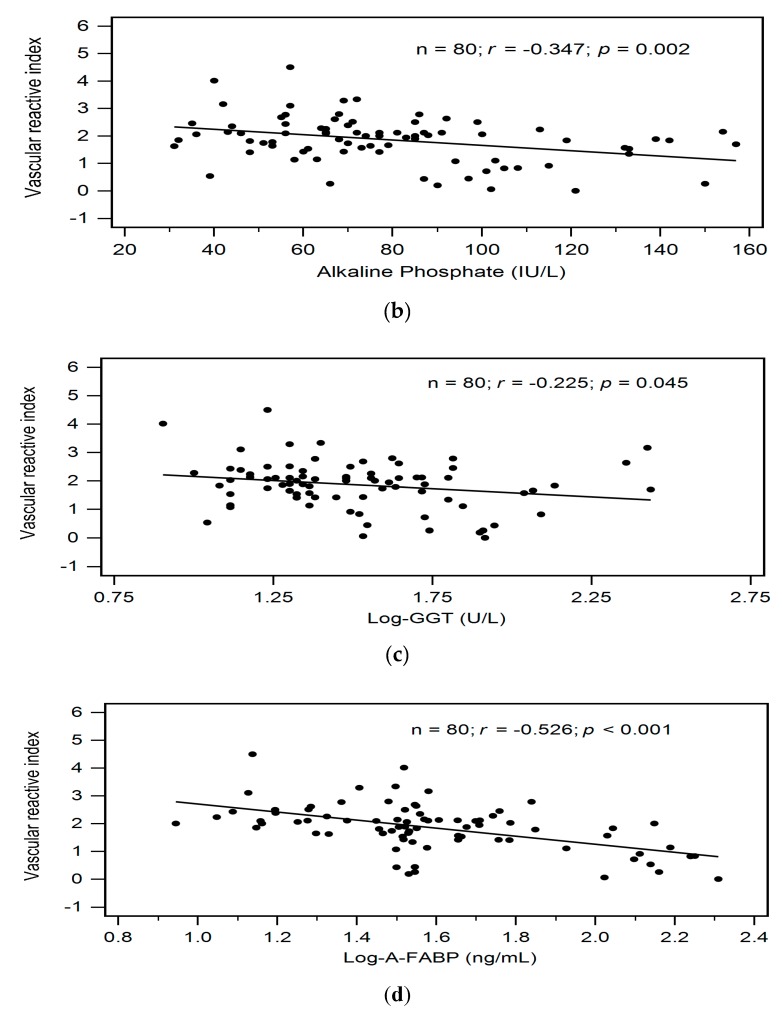
Relationships between vascular reactive index and (**a**) Age (years), (**b**) Alkaline phosphatase level (IU/L), (**c**) Log-GGT level (U/L), or (**d**) Log-A-FABP level (ng/mL) among 80 KT patients.

Correlations between clinical variables and serum VRI values determined by simple linear regression analysis and multivariable forward stepwise regression analysis of the 80 KT patients are presented in Table 2. According to simple linear regression analysis, advanced age (*r* = −0.305, *p* = 0.006), serum ALP level (*r* = −0.347, *p* = 0.002), log-transformed GGT level (*r* = −0.225, *p* = 0.045), and serum log-transformed A-FABP level (*r* = −0.526, *p* < 0.001) were negatively correlated with VRI values in KT patients. Furthermore, after adjusting with the variables that were significantly associated with VRI values, advanced age (β = −0.283, adjusted R^2^ change = 0.072, *p* = 0.003), and high serum log-transformed A-FABP level (β = −0.514, adjusted R^2^ change = 0.268, *p* < 0.001) were significantly and independently associated with VRI values in KT patients as determined by multivariable forward stepwise linear regression analysis. To better visualize the results, two-dimensional scattered plots of VRI values with age, serum ALP level, log-GGT level, and log-A-FABP level among these KT patients were drawn, which are presented as Figure 1a–d, respectively.

## 3. Discussion

This study showed that advanced age and serum levels of ALP, GGT, and A-FABP were negatively associated with VRI values measured by digital thermal monitoring in KT patients. Furthermore, after adjusting with multivariable linear regression analysis, there was a significant and independent association between age or serum level of A-FABP and VRI values in KT patients.

The endothelium is a target of injury during or after transplantation. Endothelial dysfunction can be measured by serum or urinary biomarkers, which can also indicate CV health and predict mortality in KT patients [2,3]. Over the past two decades, there have been many attempts at developing convenient non-invasive methods to measure endothelial function, including fingertip arterial tonometry, fingertip photoplethysmography, and ultrasound imaging of brachial flow-mediated dilatation [4,6]. Naghavi et al. measured vascular reactivity using digital thermal monitoring and found that advanced age and elevated diastolic blood pressure (DBP) can predict endothelial dysfunction; further, they noted that this finger-based method of measuring vascular reactivity provides a reasonable clinical assessment, which is inexpensive and convenient [5]. Flow-mediated dilatation is significantly reduced in smokers and adults with coronary artery disease, which is indicative of endothelial dysfunction [6]. Changes in endothelial function before and after KT have also been measured by endothelium-dependent vasodilation method, as assessed by reactive hyperemia [1,16]. In the current study, we similarly used a non-invasive method named digital thermal monitoring to assess endothelial function in KT patients.

A-FABP is one of the aberrantly secreted adipokines by adipose tissues, and it is overproduced in adipose tissues in obese individuals [17,18]. Moreover, abnormal overproduction of A-FABP plays a critical role in obesity-related CVDs and endothelial dysfunction by increasing the accumulation of cholesterol and triglyceride and enhancing inflammation; together, this results in insulin resistance, atherosclerosis, and impaired activation of endothelial nitric oxide synthase (eNOS) [19]. In women with gestational diabetes, serum A-FABP level is independently correlated with impaired glucose metabolism and increases the risk of progression to DM [20]. In addition, higher levels of circulating A-FABP are believed to be a biomarker for predicting unfavorable CV events in patients with coronary artery disease, end-stage renal disease, or critical illness [13,21,22]. In ApoE-deficient mice and in the human microvascular endothelium, previous work shows that A-FABP has an independent role in atherogenesis; additionally, enhanced expression of A-FABP was associated with reduced expression of eNOS and nitric oxide (NO) production [19,23]. Additional work showed an association between serum A-FABP levels and endothelial dysfunction [24,25,26]. Furuhashi et al. reported that serum levels of A-FABP correlated positively with changes in carotid intima-media thickness in subjects without medications [25]. Aragones et al. demonstrated that circulating A-FABP levels were associated with endothelial dysfunction as measured by reactive hyperemia index in DM patients [24]. Moreover, the role of A-FABP in inhibiting eNOS activation and NO production through the decreased expression of insulin receptor substrate 1 and Akt was later verified in vitro [26]. In this study, we demonstrated a significantly negative correlation between serum A-FABP levels and VRI in KT patients. This finding indicates that A-FABP plays a role in modulating endothelial dysfunction in KT patients.

Among all possible factors in the development of endothelial dysfunction, advanced age plays an important role [4]. Age-associated endothelial dysfunction, as most commonly observed by impaired endothelium-dependent dilation, is mediated by reduced NO bioavailability and increased production of reactive oxygen species in vivo [27]. In healthy men, higher expression and bioactivity of vasoconstrictors such as endothelin-1 but not vasodilators such as eNOS, contribute to impaired endothelial dysfunction with aging [28]. Furuhashi et al. found that aging was an independent predictor of carotid intima-media thickness in a population cohort study, with an inverse correlation between VRI and age in another large patient registry [5,25]. In the present study, we similarly found that advanced age was inversely correlated with VRI values in KT patients.

Throughout population discovery and experimental findings, recent studies proposed the role of ALP and GGT in CVD development [29,30]. Kunutsor et al. showed in a meta-analysis that there was a positive linear correlation between serum ALP and GGT levels and risk of CVDs in the general population [29]. For GGT, this was reported to be associated positively with all-cause mortality and CVD through possible mechanisms of pro-oxidant and pro-inflammatory activities as well as having a role in atheromatous plaque formation [29]. Additionally, Li et al. found in a population of preserved renal function patients a significant relationship between ALP levels and events of coronary artery diseases, CVDs, and death, especially in those with DM and CVD [30]. Moreover, there was a significant inverse correlation between serum ALP levels and endothelium-dependent dilatation in hypertensive naïve patients [31]. Taken together with these studies, our data showing serum ALP and GGT level inversely correlated with VRI in KT patients suggests a role of ALP in modulating endothelial function.

This study has some limitations, which warrant discussion. First, this was a cross-sectional study conducted at a single center, with a limited number of KT patients. Second, our study did not measure microalbuminuria or proteinuria, although there were studies reported that serum A-FABP levels are positively associated with microalbuminuria in newly diagnosed type 2 DM patients [32] and microalbuminuria or proteinuria is one of the risk factors for progression of CKD in KT patients [33]. Future studies should employ a longitudinal method to determine a causal relationship between serum A-FABP levels and endothelial function in a larger cohort.

In conclusion, to the best of our knowledge, this is the first study to demonstrate that fasting A-FABP level was negatively associated with VRI, which is representative of endothelial function as measured by digital thermal monitoring in KT patients.

## 4. Materials and Methods

### 4.1. Participants

In total, we examined 80 KT patients enrolled at a medical center in Hualien, Taiwan, from September 2015 to February 2016. Patients who had systolic blood pressure ≥ 140 mmHg and/or diastolic blood pressure ≥ 90 mmHg or were receiving any anti-HTN medications in the previous 2 weeks were diagnosed with HTN. Patients were diagnosed with DM if their fasting plasma glucose level was either ≥ 126 mg/dL or if they were using oral hypoglycemic medications or insulin. Status of smoking and usage of medications were reviewed by medical records. Patients were excluded if they had an acute infection, acute myocardial infarction, heart failure, acute transplant rejection status, or malignancy at the time of blood sampling. The Protection of the Human Subjects Institutional Review Board of Tzu-Chi University and Hospital approved this study (IRB104-27-B). All patients provided written informed consent before participating in this study.

### 4.2. Anthropometric Analysis

Patient body weight was measured to the nearest 0.5 kg and body height to the nearest 0.5 cm, while wearing light clothing and without shoes. Body mass index (BMI) was calculated as weight in kg divided by height in meters squared. 

### 4.3. Biochemical Investigations

Fasting blood samples (approximately 5 mL) of all patients were immediately centrifuged at 3000× *g* for 10 min and then were tested for serum levels of blood urea nitrogen, creatinine, glucose, total cholesterol, triglycerides, high-density lipoprotein cholesterol (HDL-C), calcium, phosphorus, GGT, and ALP using an auto-analyzer (Siemens Advia 1800, Siemens Healthcare GmbH, Henkestr, Germany). The estimated glomerular filtration rate (eGFR) was calculated by the CKD-EPI (Chronic Kidney Disease Epidemiology Collaboration) equation. Serum A-FABP and intact parathyroid hormone (iPTH) levels were measured using a commercially available enzyme immunoassay (EIA; SPI-BIO, Montigny-le-Bretonneux, France) and enzyme-linked immunosorbent assay (Diagnostic Systems Laboratories, Webster, TX, USA), respectively. 

### 4.4. Endothelial Function Measurements

After overnight fasting, and resting in a supine position in an ambient temperature of 22–24 °C for 30 min, digital thermal monitoring of endothelial function was performed by utilizing a computer-based thermometry system of an FDA-approved device (VENDYS-Ⅱ, Endothelix Inc., Houston, TX, USA). Standard sphygmomanometer cuffs were placed on right upper arm of the subject. Skin temperature sensors were affixed to both index fingers of the subject (left: control; right: occlusion). Digital thermal monitoring of both hands was obtained during 5-minute stabilization, 5-minute cuff inflation, and 5-minute deflation using an automated, operator independent protocol. The right upper arm cuff (i.e., occluded arm) was rapidly inflated to 50 mmHg greater than systolic blood pressure for 5 min and then rapidly deflated to invoke reactive hyperemia distally. Thermal changes during a 5-minute arm-cuff-induced reactive hyperemia test were monitored continuously in the fingertip of both the occluded (i.e., right) and the non-occluded (i.e., left) arms using VENDYS software. During the cuff occlusion, fingertip temperature in the right index finger falls because of the absence of warm circulating blood. The occlusion of blood flow elicits a vasodilatory response in the ischemic area. Once the cuff is released, blood flows into the forearm and hand, causing a temperature rebound in the fingertip of right index finger, which is directly proportional to the reactive hyperemia response. Finally, the area under the temperature curve was used to determine the VRI by taking the maximum difference between the observed temperature rebound curve and the zero-reactivity curve during the reactive hyperemia period [5]. Patients were defined as having poor, intermediate, or good VR if VRI was <1.0, 1.0–1.9, or ≥2.0, respectively [5].

### 4.5. Statistical Analysis

Data are expressed as means ± standard deviation (SD), and normal distribution was determined by Kolmogorov–Smirnov test. Differences among groups (poor, intermediate, and good VRI) were analyzed using Kruskal–Wallis analysis for parameters not normally distributed or a one-way analysis of variance (ANOVA) for normally distributed data followed by the Fisher’s protected *t* test. Since the distributions of fasting glucose, triglyceride, blood urea nitrogen, creatinine, GGT, iPTH, and A-FABP levels were skewed, data were then log-transformed to achieve normality. Variables correlated with VRI values were evaluated by simple linear regression analyses; those variables significant in the simple linear regression analyses were further analyzed by a multivariable forward stepwise regression analysis. Data were analyzed by using SPSS for Windows (version 19.0, SPSS Inc., Chicago, IL, USA). The value *p* < 0.05 was considered statistically significant. 

## Figures and Tables

**Table 1:** Clinical characteristics according to different vascular reactivity index measured by digital thermal monitoring of 80 KT patients.

Characteristics	All Patients (*n* = 80)	Good Vascular Reactivity (*n* = 38)	Intermediate Vascular Reactivity (*n* = 30)	Poor Vascular Reactivity (*n* = 12)	*p* Value
Age (years)	45.29 ± 10.61	45.03 ± 11.32	46.97 ± 9.80	48.58 ± 10.57	0.549
KT duration (months)	74.80 ± 51.50	70.87 ± 44.79	87.37 ± 55.15	55.83 ± 58.39	0.163
Height (cm)	161.39 ± 7.57	160.59 ± 6.96	161.40 ± 7.27	163.88 ± 10.00	0.429
Body weight (kg)	64.35 ± 13.16	64.80 ± 13.33	64.40 ± 12.71	62.79 ± 14.72	0.901
Body mass index (kg/m^2^)	24.65 ± 4.48	25.13 ± 5.08	24.60 ± 3.63	23.24 ± 4.45	0.448
Vascular reactivity index	1.87 ± 0.84	2.51 ± 0.56	1.61 ± 0.26	0.46 ± 0.31	<0.001 *
SBP (mmHg)	144.43 ± 19.00	143.05 ± 18.19	145.37 ± 21.71	146.42 ± 14.93	0.821
DBP (mmHg)	83.66 ± 12.51	84.42 ± 13.25	82.80 ± 12.73	83.42 ± 10.13	0.869
Total cholesterol (mg/dL)	189.94 ± 46.41	183.32 ± 37.72	200.40 ± 56.47	184.75 ± 42.40	0.298
Triglyceride (mg/dL)	127.00 (90.25–192.75)	114.50 (85.50–198.25)	128.50 (96.00–186.50)	155.00 (99.25–194.25)	0.566
HDL-C (mg/dL)	49.95 ± 15.48	49.55 ± 15.13	51.27 ± 16.63	47.92 ± 14.58	0.803
Fasting glucose (mg/dL)	96.00 (88.25–113.75)	95.50 (87.00–107.50)	99.50 (88.75–134.50)	94.00 (87.50–122.50)	0.379
Blood urea nitrogen (mg/dL)	24.00 (16.00–37.00)	23.50 (16.75–29.75)	26.00 (15.50–42.75)	27.00 (15.00–38.50)	0.893
Creatinine (mg/dL)	1.40 (1.00–1.90)	1.30 (1.00–1.73)	1.60 (0.98–2.30)	1.35 (1.15–1.83)	0.342
eGFR (mL/min)	56.17 ± 25.91	59.95 ± 22.66	52.35 ± 29.68	57.75 ± 23.55	0.596
Total Calcium (mg/dL)	9.14 ± 0.69	9.13 ± 0.66	9.12 ± 0.75	9.20 ± 0.66	0.938
Phosphorus (mg/dL)	3.36 ± 0.81	3.33 ± 0.80	3.41 ± 0.87	3.35 ± 0.73	0.925
Alkaline phosphate (IU/L)	79.13 ± 29.99	70.39 ± 24.38	82.47 ± 34.61	98.42 ± 27.61	0.012 *
GGT (U/L)	30.00 (20.00–52.00)	24.50 (17.00–42.50)	23.00 (20.00–52.25)	54.00 (33.25–81.75)	0.032 *
A-FABP (ng/mL)	34.34 (26.13–54.14)	30.80 (17.30–37.93)	35.07 (31.91–57.95)	127.02 (35.11–166.03)	<0.001 *
iPTH (pg/mL)	101.45 (57.28–160.65)	91.15 (56.08–144.93)	116.80 (59.75–173.00)	90.50 (42.25–179.65)	0.477
Female, *n* (%)	40 (50.0)	22 (57.9)	14 (46.7)	4 (33.3)	0.299
Diabetes mellitus, *n* (%)	39 (48.8)	18 (47.4)	16 (53.3)	5 (41.7)	0.770
Hypertension, *n* (%)	31 (38.8)	11 (28.9)	15 (50.0)	5 (41.7)	0.205
Living donor, *n* (%)	16 (20.0)	4 (10.5)	9 (30.0)	3 (25.0)	0.123
Statin use, *n* (%)	33 (41.2)	13 (34.2)	14 (46.7)	6 (50)	0.468
Smoking, *n* (%)	6 (7.5%)	1 (2.6)	3 (10)	2 (16.7)	0.221
Tacrolimus use, *n* (%)	53 (60.3)	26 (68.4)	20 (66.7)	7 (58.3)	0.811
MMF use, *n* (%)	48 (60.0)	22 (55.9)	19 (63.3)	7 (58.3)	0.895
Steroid use, *n* (%)	68 (85.0)	32 (84.2)	26 (86.7)	10 (83.3)	0.946
Rapamycin use, *n* (%)	8 (10.0)	2 (5.3)	5 (16.7)	1 (8.3)	0.291
Cyclosporine use, *n* (%)	14 (17.5)	8 (21.1)	3 (10.0)	3 (25.0)	0.374

Values for continuous variables given as means ± standard deviation and test by one-way analysis of variance; variables not normally distributed given as medians and interquartile range and test by Kruskal–Wallis analysis; values are presented as number (%) and analysis after analysis by the chi-square test. KT, kidney transplant; SBP, systolic blood pressure; DBP, diastolic blood pressure; HDL-C, high-density lipoprotein cholesterol; eGFR, estimated glomerular filtration rate; GGT, γ-glutamyltranspeptidase; A-FABP, adipocyte fatty acid-binding protein; iPTH, intact parathyroid hormone; MMF, mycophenolate mofetil. * *p* < 0.05 was considered statistically significant after Kruskal–Wallis analysis or one-way analysis of variance.

**Table 2:** Correlation of vascular reactivity index levels and clinical variables by simple or multivariable linear regression analyses among 80 KT patients.

Variables	Vascular Reactivity Index				
	Simple Linear Regression		Multivariable Linear Regression		
	*r*	*p* Value	Beta	Adjusted R^2^ Change	*p* Value
Female	0.170	0.131	-	-	-
Diabetes mellitus	−0.034	0.765	-	-	-
Hypertension	−0.183	0.104	-	-	-
Living donor	−0.111	0.329	-	-	-
Statin use	−0.159	0.159	-	-	-
Smoking	−0.145	0.199	-	-	-
Age (years)	−0.305	0.006 *	−0.283	0.072	0.003 *
KT duration (months)	−0.093	0.412	-	-	-
Height (cm)	−0.138	0.224	-	-	-
Body weight (kg)	−0.034	0.764	-	-	-
Body mass index (kg/m^2^)	0.038	0.740	-	-	-
Systolic blood pressure (mmHg)	−0.160	0.156	-	-	-
Diastolic blood pressure (mmHg)	0.046	0.683	-	-	-
Total cholesterol (mg/dL)	−0.096	0.397	-	-	-
Log-Triglyceride (mg/dL)	−0.205	0.069	-	-	-
HDL-C (mg/dL)	0.079	0.487	-	-	-
Log-Glucose (mg/dL)	−0.155	0.169	-	-	-
Log-Blood urea nitrogen (mg/dL)	−0.111	0.329	-	-	-
Log-Creatinine (mg/dL)	−0.084	0.458	-	-	-
eGFR (mL/min)	0.082	0.470	-	-	-
Total Calcium (mg/dL)	−0.013	0.907	-	-	-
Phosphorus (mg/dL)	0.001	0.992	-	-	-
Alkaline phosphate (IU/L)	−0.347	0.002 *	-	-	-
Log-GGT (U/L)	−0.225	0.045 *	-	-	-
Log-A-FABP (ng/mL)	−0.526	<0.001 *	−0.514	0.268	<0.001 *
Log-iPTH (pg/mL)	0.033	0.775	-	-	-

Data of triglyceride, fasting glucose, blood urea nitrogen, creatinine, GGT, iPTH, and A-FABP showed skewed distribution and therefore were log-transformed before analysis. Analysis of data was done using the simple linear regression analyses or multivariable stepwise linear regression analysis (adapted factors were age, alkaline phosphate, log-GGT, and log-A-FABP). KT, kidney transplant; HDL-C, high-density lipoprotein cholesterol; eGFR, estimated glomerular filtration rate; GGT, γ-Glutamyltranspeptidase; A-FABP, adipocyte fatty acid-binding protein; iPTH, intact parathyroid hormone. * *p* < 0.05 was considered statistically significant.
